# Structure and Dynamics of Water Confined at the SiO_2_/WS_2_ Interface

**DOI:** 10.1021/acs.jpcc.4c08392

**Published:** 2025-02-17

**Authors:** Katherine L. Milton, Laura Hargreaves, Alexander Shluger

**Affiliations:** † Department of Physics and Astronomy and the London Centre for Nanotechnology, University College London, Gower Street, London WC1E 6BT, U.K.; ‡ WPI-Advanced Institute for Materials Research (WPI-AIMR), Tohoku University, 21-1 Katahira, Aoba-ku, Sendai 980-8577, Japan

## Abstract

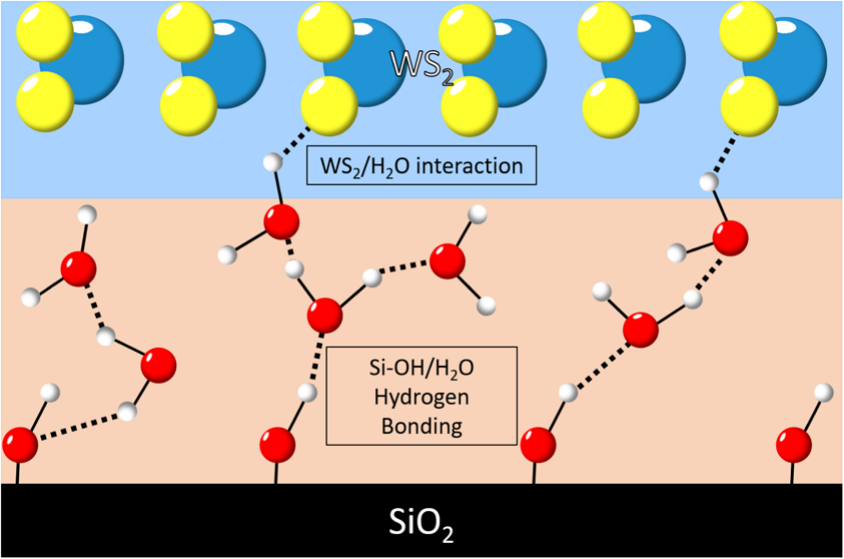

The WS_2_/SiO_2_ interface is of interest to a variety of research communities due to the electronic properties of WS_2_ and the ubiquity of SiO_2_ as a dielectric substrate. Due to the hydrophilic nature of silanol groups on the surface of SiO_2_, water is difficult to remove at the surface, leading to confined water between WS_2_ and SiO_2_. Understanding the properties of confined water is important both fundamentally and for their effects on the interfacing materials. We investigated the structure and dynamics of confined water between WS_2_ and SiO_2_ using density functional theory and *ab initio* molecular dynamics, comparing it to adsorbed water on the surfaces of WS_2_ and SiO_2_. The results show that confined water becomes increasingly structured, with its orientation influenced by hydrogen bonding to the silanol groups as well as by the partial reorientation of water molecules to face WS_2_ in an H-up configuration. The presence of silanol groups disrupts the hydrogen bonding network of water at monolayer coverage for both confined and unconfined water. For all interfaces explored, changes in both structural and dynamic properties are dependent on the number of water layers present.

## 1 Introduction

Studies and applications of WS_2_ and other transition metal dichalcogenides (TMDs) often require growth or deposition of thin films of these 2D materials on different substrates.^1−3^ Insulating substrates, such as Al_2_O_3_, SiO_2_, and h-BN, are often used for potential applications of these heterostructures in microelectronics, sensing and catalysis. From this perspective, understanding how the interaction of TMDs with substrates affects the properties of each material and the performance of the heterostructure is of great interest. In this work, we are particularly focused on the WS_2_/SiO_2_ interface, but the issues we discuss are common to other TMDs deposited on SiO_2_ substrates.

Without special treatment, silica surfaces exposed to humidity are covered by Si–OH (silanol) groups, resulting in hydrophilicity of the surface and the presence of water at ambient temperatures and pressures.^4^ The hydrogen bonding of water to the silanol groups at the silica surface causes the water to reorient and reduce mobility near the surface, although the exact structure of the water is still controversial.^5,6^ The deposition of a WS_2_ film is likely to trap a certain amount of water between SiO_2_ and WS_2_ surfaces. The structure and dynamics of this nanoconfined water is the main focus of this study.

The silanol groups on the SiO_2_ surface can significantly influence the water structure near the interface, primarily through hydrogen bonding (HBonding) between the silanol groups and water molecules. The density and amount of water at the interface can further affect the structural and dynamic properties of the system. However, the exact structural changes induced by the silanol groups remain a subject of debate in the literature.^7^

Infrared (IR) Attenuated Total Reflection (ATR) studies suggest that, at relative humidity (RH) levels below 50%, water forms ice-like layers that are strongly hydrogen bonded. Above 50% RH, water transitions to more disordered, liquid-like structures.^5^ Theoretical studies using AIMD have also demonstrated that near the silica surface, where silanol groups are present, water forms ice-like layers connected by HBonds.^8,9^

However, heterodyne-detected (HD)-VSFG experiments suggest that at 20% RH, nanodroplets begin to form and between 20% and 50% RH, partially liquid water layers emerge, becoming uniform by 90% RH.^6^ The formation of these nanodroplets is attributed to preferential interactions between water and silanol groups. Similar results were observed in a separate study using IR-ATR.^10^ Further work with nonlinear SFG has suggested the formation of two different layers: the binding interfacial layer (BIL), and the diffuse layer (DL). The BIL is tightly hydrogen bonded to the silanol groups, and the diffuse layer is much less structured but does show some reorientation of the water molecules due to stable HBonds formed with the BIL.^11−13^ Therefore, one can expect that water is tightly HBonded to the silanol groups on the SiO_2_ surface. However, the transition point at which water becomes bulk water is still undefined.

On the other side of the interface, the WS_2_ water contact angle (WCA) measurements exhibit considerable variations, ranging from 38.8 to 90°. These variations are influenced by factors such as substrate type, TMD thickness, air exposure, and surface oxidation.^14−21^ While these factors substantially impact the hydrophilicity of WS_2_, studies of pristine WS_2_ indicate that it exhibits mild hydrophilicity, with a WCA of approximately 70°.^14,18^ The interaction between TMDs and water is strong enough to support their use in water-sensing devices.^22^

Classical molecular dynamics (CMD) simulations have provided deeper insights into the behavior of water on TMD surfaces. For example, polygonal water clusters have been observed on MoS_2_ and WSe_2_ surfaces with bulk water.^23−25^ The structuring of water predominantly occurs laterally to promote maximum HBonding among water molecules. When TMDs are exposed to significant amounts of water, CMD simulations predict the dewetting of water on MoS_2_. This process leads to the formation of droplets.^26^ Further exposure of MoS_2_ to liquid water during experiments results in its oxidation to MoO_3_·H_2_O, which forms needle-like structures.^20^

Beyond interactions with water at the surface, recent studies have explored water confined between two two-dimensional (2D) sheets to understand the structures and interactions that emerge in these confined environments.^27^ The application of 2D materials for the confinement of water has been studied in desalination, focusing on graphene and TMDs MoS_2_ and WS_2_.^28^

Branching out from 2D material confinement, the choice of confining substrate can significantly influence the structure, reactivity, and properties of water. For example, water confined between Ru and SiO_2_ has a reduced activation energy for the oxidation of H_2_.^29^ Investigations of water confined between graphene and substrates, such as SiO_2_ or mica, reveal the formation of ice-like double-layer structures, which allows water to diffuse between layers.^30^ Furthermore, graphene and mica display ice-like water layers with a thickness of 3.7 Å, with structural defects in graphene playing a significant role in water adsorption.^27,31^ Similar behavior has been observed in MoS_2_/mica systems, where atomic force microscopy (AFM) studies have demonstrated unique frictional properties and morphological deformation of MoS_2_ in the presence of silica.^32,33^ Water intercalation and the formation of water nanostructures have also been noted in MoS_2_/mica interfaces under varying humidity conditions.^34^

However, the impact of SiO_2_ substrates that introduce HBonding with confined water has not been thoroughly examined. To our knowledge there has been no previous investigation into the confined water at the SiO_2_/WS_2_ interface although water can be easily trapped between the two surfaces when the 2D material is mechanically or chemically exfoliated onto SiO_2_.^35^ Therefore, the SiO_2_/WS_2_ interface provides a natural platform to investigate the effects of HBonding on confined water. Previous studies of confined water between 2D materials and dielectric oxides have primarily used mica due to its ease of cleavage and low surface roughness. In contrast, SiO_2_ has a much rougher surface, making it difficult to study using scanning probes. Therefore, understanding this interface heavily relies on theoretical modeling.

We investigate the structure and dynamics of water between SiO_2_ and WS_2_ by analyzing and comparing the structural and dynamic properties of the SiO_2_/H_2_O/WS_2_, SiO_2_/H_2_O, and WS_2_/H_2_O interfaces. We employ *ab intio* molecular dynamics (AIMD) and simulate systems containing one, two, and three layers of water. The water consistently exhibits a long-range structure induced by silanol groups on the SiO_2_ surface, while a double-layer structure is observed at the WS_2_ interface. At both the WS_2_ and SiO_2_ interfaces, the hydrogen atoms of water are drawn closer to the surfaces; this effect is amplified with the introduction of confinement. While AIMD offers a higher level of accuracy, it has certain limitations, most notably restricting simulation time scales to picoseconds (ps), compared to the nanosecond (ns) range achievable with CMD. As a result, some dynamic properties may be affected due to the limited sampling. Nonetheless, AIMD provides accurate modeling of the system, serving as a reliable foundation for future studies, such as training machine learning potentials (MLP) or providing a benchmark for other computational methods.

## 2 Methodology

Since the interaction of water with silanol groups is an important factor in determining the structure of confined water, we chose the (101̅) surface of the SiO_2_ crystalline phase α-cristobalite (α-C) to represent the silica structure. It is known to be a good mimic of the density and distribution of single silanol groups on an amorphous silica surface.^36^ The density of silanol groups on this surface (4.7 OH nm^–2^) is similar to that of amorphous silica (4.5 OH nm^–2^), which is predominantly used as a substrate. We therefore use α-C as a model substrate in all further calculations. Based on previous simulations of the properties of silica surfaces, we expect that disorder in locations of silanol groups on amorphous surfaces will not affect our conclusions.^37,38^ The bulk structure of 3 × 3 × 3 α-cristobalite (α-C) SiO_2_ was optimized using the CP2K^39^ code with the double-ζ Gaussian basis sets,^40^ the GTH pseudopotential,^41^ and the PBE0-TC-LRC exchange functional. A 101̅ plane of α-C was cut and a 231 super cell was produced.

CMD was used to add and equilibrate water on the SiO_2_ surface. We used the standard 12/6 Lennard-Jones potential with parameters given by the INTERFACE force field^36^ for the Si, O, and H atoms in silica and the TIP4*P*/2005f force field^42^ for the H and O atoms in water. Geometric mixing rules were applied to the interactions between water and silica. After achieving equilibrium, the amount of water selected was based on the radial distribution function and the water was truncated to reduce the amount of water to 1, 2, and 3 water layers. The truncated system was then re-equilibrated using CMD.

**Figure 1:**
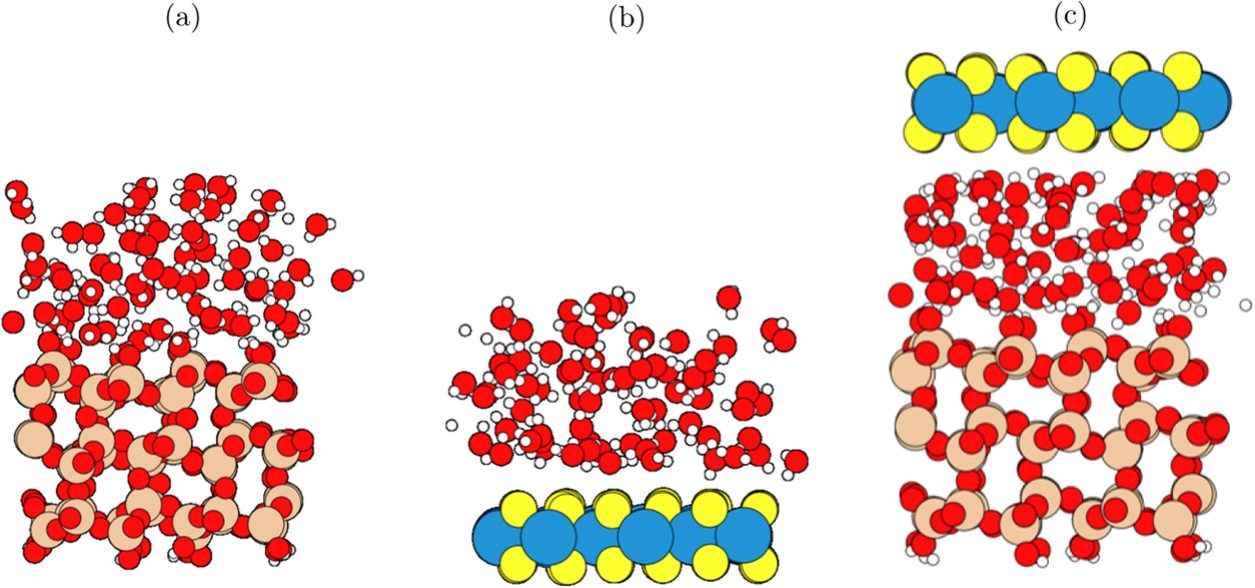
Snapshot geometric structures used in this work, with three water layers shown for all interface structures. (a) Shows the SiO_2_/H_2_O interface, (b) shows the WS_2_/H_2_O interface, and (c) shows the SiO_2_/H_2_O/WS_2_ interface. For every interface, there is an extended vacuum region above and below, not shown in this figure. colors: blue = W, yellow = S, red = O, white = H, beige = Si.

A 5 × 3 orthorhombic WS_2_ monolayer was added to the CMD optimized silica–water truncated interface. This system was used in AIMD calculations using the PBE-D3 XC functional, which is reliable for SiO_2_/H_2_O systems.^43^ An NVT setup maintained at 400 K with a Nosé–Hoover thermostat was used to mitigate the overbinding of water with PBE-D3, as discussed in ref (44). All AIMD calculations were run for 4 ps where a pseudoequilibrium was reached, as the system’s total energy remained constant. The production run was then taken over a 12.5 ps run time. Three systems were tested: 1-layer, 2-layers, and 3-layers of water with AIMD with snapshots of each system shown in Figure 1. The total number of atoms in the confined system was: 396 (9 water molecules), 444 (34 water molecules) and 558 (72 water molecules) for each water layer system. Further details of this setup are described in ref (44).

### 2.1 Geometric Analysis

Given that water was included in the simulations, the geometric configurations of the system evolve over time. Variations in the positions of water molecules, influenced by factors such as the presence of silanol groups and the number of water molecules, were analyzed using the MDAnalysis package.^45,46^

The one-dimensional (1D) density profile calculates the spatial distribution of water molecules. Additionally, further selection criteria can be applied to look at the water hydrogen (H_*w*_) and/or water oxygen (O_*w*_) atoms. The 1D density is defined as
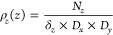
1where *N*_*z*_ is the number of molecules, *D* is the dimension of the cell in the respective direction, and δ_*z*_ is the grid spacing which was set to 0.1 Å. The density is calculated for every frame in the trajectory and averaged. The results allow for the investigation of water layering in the z direction and the specific arrangement of H_*w*_ and O_*w*_. To enable comparison between the various water layers at each interface, the density was normalized to 1.

The joint probability distribution (JPD) provides a good description of the water orientation by combining the probability of two different angles. The JPD of the water angles were calculated by aggregating two angles into a 2D histogram and normalized to get the probability as the total sum of all bins is one. The OH–OH bond angle was analyzed in this work following the work of Smirnov^13^ to ensure that the orientation of water could be determined. Since the distribution of correlated angles may be broad, we will focus on the orientations derived from the midpoint of the distribution, where the probability is highest.

Hydrogen bonds between water molecules were characterized based on geometric criteria, specifically the distance and angle between neighboring molecules. The cutoff distance for HBond formation was defined as 1.2 Å, with a maximum distance between the donor and acceptor oxygen set at 3.0 Å. The cutoff for the O–H–O angle was set to 150°.

Continuous hydrogen bond lifetimes were analyzed using the autocorrelation function^47^
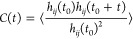
2where *h*_*ij*_ represents the existence of a hydrogen bond between atoms *i* and *j*, with *h*_*ij*_ = 1 if a bond is present and *h*_*ij*_ = 0 otherwise. At the initial time, *h*_*ij*_(*t*_0_) is equal to one. The calculation is averaged over multiple starting times *t*_0_ to improve statistical reliability.

The HBond lifetimes were calculated by fitting a biexponential curve to the autocorrelation using the form
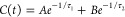
3where *A* and *B* are the pre-exponential factors and sum to 1, and both τ_1_ and τ_2_ are the time constants, and τ_1_ is for short-time scale processes and τ_2_ is longer time scale processes. This description of the autocorrelation then allows for the HBond lifetime (τ) to be calculated using
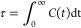
4

The mean squared displacement (MSD) of water molecules was computed using the Einstein relation
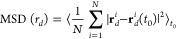
5where *N* is the total number of particles in the system, **r**^*i*^ are the coordinates of each particle, d is the system dimensionality, *t*_0_ is the initial time. From this we can calculate the self-diffusivity
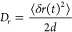
6

To obtain ⟨*δr*(*t*)^2^⟩ we applied a linear fit to the MSD data.

## 3 Results and Discussion

### 3.1 Static Properties

We begin by calculating the static properties of the system, focusing on the average positions and orientations of water molecules at each interface.

#### 3.1.1 SiO_2_/H_2_O Interface

We first examine the water structure at the SiO_2_/H_2_O interface. The 1D density profile of water hydrogen (H_*w*_) (Figure 3a) shows that all water layers exhibit a distinct initial peak between 2.0–2.4 Å, followed by a water oxygen (O_*w*_) peak at 2.5–2.7 Å Figure 3b. The presence of a sharp peak demonstrates significant structuring at the interface, with H_*w*_ generally closer to the SiO_2_ surface than O_*w*_.

Farther from the surface, the final peak in the three layers is a small O_*w*_ peak at 7.3 Å, while the water density continues to 12 Å. This observation indicates a more liquid-like structure of water at greater distances from the silanol-terminated surface. The two distinct regions of water correspond to the DL (close to the surface, highly structured) and the BIL (further from the surface, less structured).

**Figure 2:**
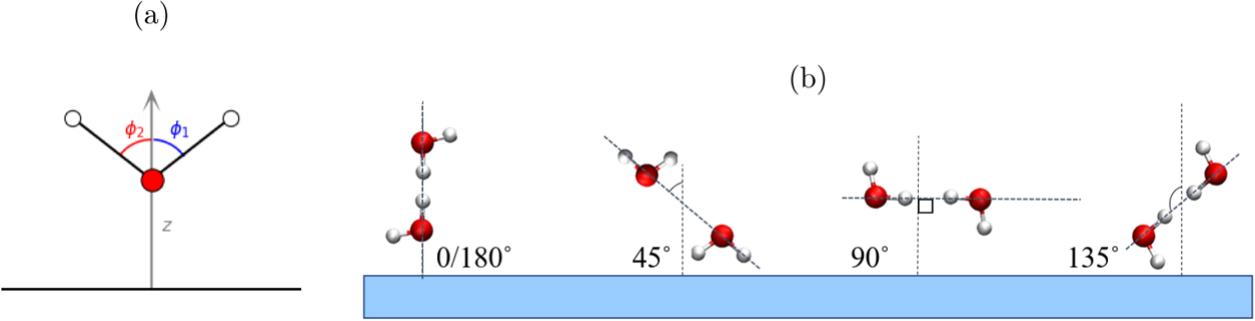
Schematics for relative angles of water relative to the surface normal of O–H orientations. (a) gives the overall calculation of both OH–OH bond angles, and (b) highlights different OH bond orientations.

To further investigate the BIL structure of water, the JPD of the SiO_2_/H_2_O one-layer interface (Figure 4a) reveals two distinct correlated angles and using Figure 2, we can deduce the orientation of water. The first, most prevalent OH–OH angle is (110°, 110°), corresponding to a predominantly flat orientation but exhibits a slight out-of-plane H_*w*_ tilt downward toward SiO_2_ the surface. The second peak is at (15°, 115°), showing a water molecule with one H_*w*_ tilted upward from the SiO_2_ surface (Figure 4b).

Overall, these 1D profiles align well with previous studies of silica–water interfaces with similar silanol densities using CMD simulations, confirming that our AIMD results are reliable for reproducing average properties.^13,48,49^

#### 3.1.2 WS2_2_/H_2_O Interface

**Figure 3:**
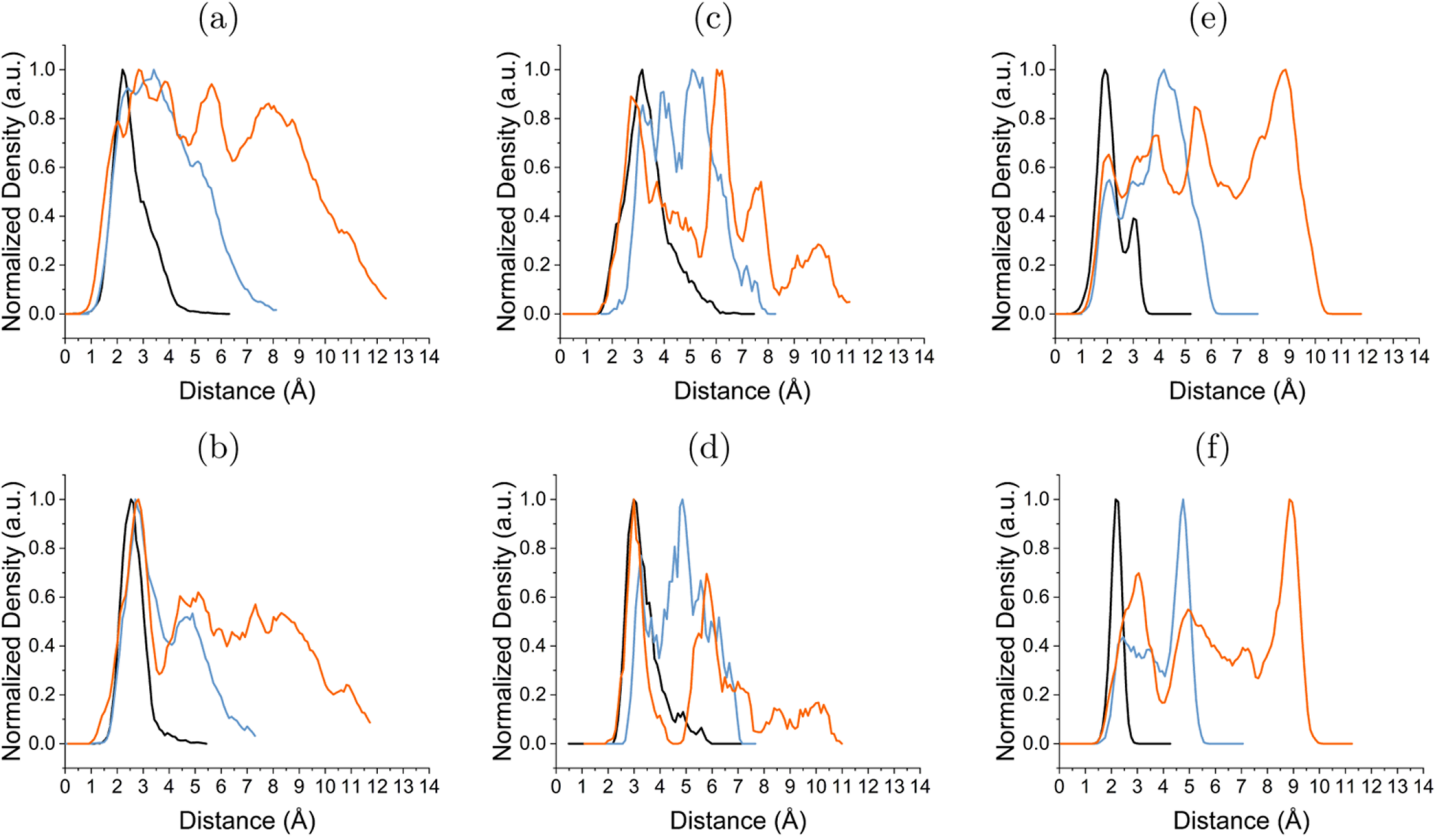
1D number density profiles of water for (a, c, e) hydrogen (H_*w*_) and (b, d, f) oxygen (O_*w*_). The interfaces shown are (a, b) SiO_2_/H_2_O, (c, d) WS_2_/H_2_O, (e, f) SiO_2_/H_2_O/WS_2_. 0 on the *x* axis corresponds to the top Si atom of the SiO_2_ surface for SiO_2_/H_2_O and SiO_2_/H_2_O/WS_2_ interfaces, and the closest sulfur plane for the WS_2_/H_2_O interface. The different water layers are given by different colors: black: one layer, blue: two layers, orange: three layers.

**Figure 4:**
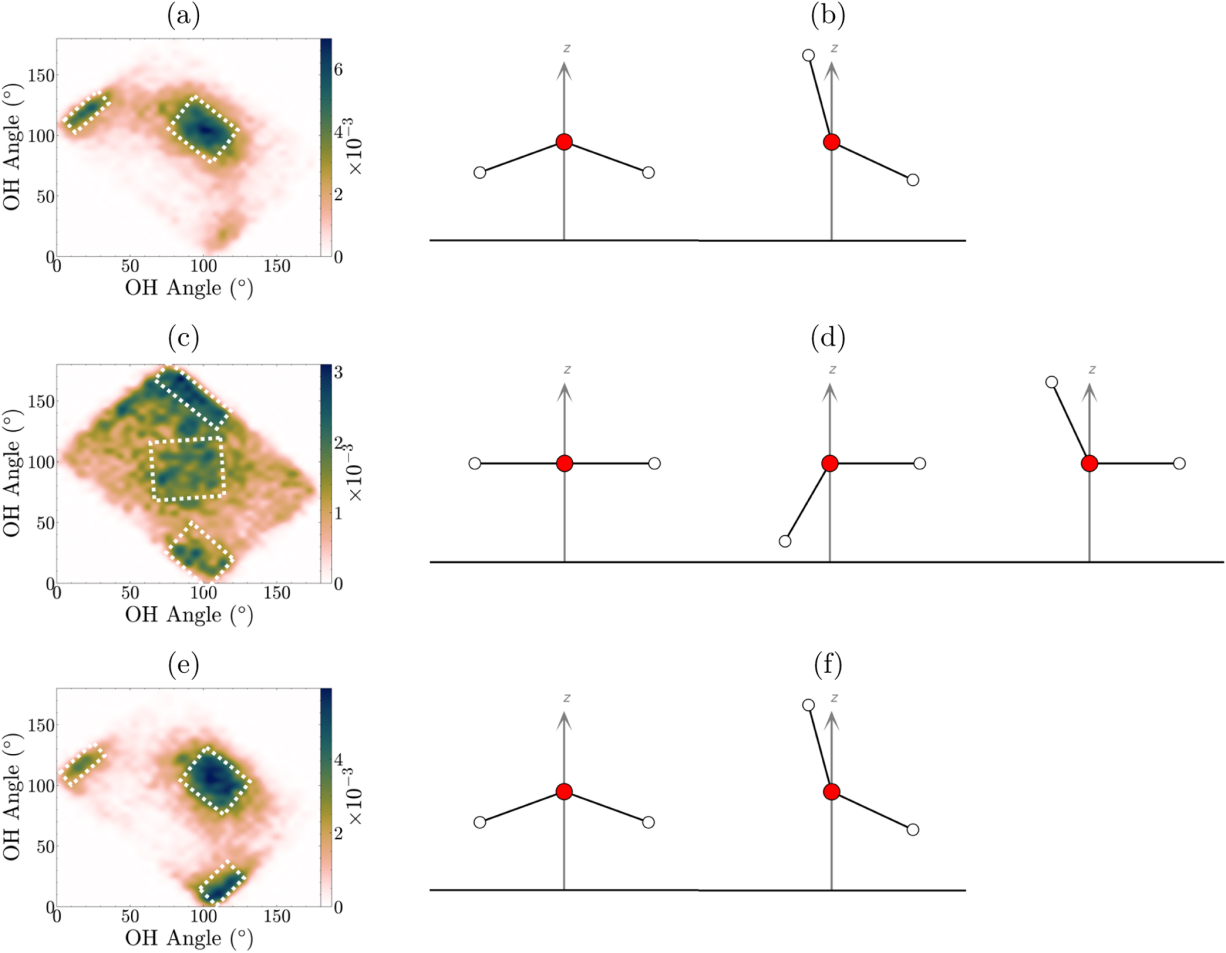
Probability distribution of OH–OH water angles for (a) SiO_2_/H_2_O, (c) WS_2_/H_2_O and (e) SiO_2_/H_2_O/WS_2_. The color bar shows the probability. The right-hand side (b, d, f) shows the corresponding water orientations, which are highlighted in the JPD graph; duplicates have been removed for clarity.

At the WS_2_/H_2_O interface, the initial O_*w*_ and H_*w*_ for all systems are centered around 2.8–3.2 Å (Figures 3a,3d). Therefore, it is expected that the water will be oriented parallel to the surface. However, for all layers there is a H_*w*_ shoulder closer to the surface at 2.0–2.2 Å, so there is a slight preference of H_*w*_ to orient toward WS_2_ (For precise details see SI Table S1). The WS_2_/H_2_O one-layer JPD indicates three distinct orientations (Figure 4c). The predominant orientation is observed at (90°, 150°), where one O–H bond is parallel to the WS_2_ surface, while the other H_*w*_ points down toward the WS_2_. The two alternative orientations are characterized by angles of (90°, 90°), where the water molecule is entirely parallel to the WS_2_ surface, and (90°, 25°) where one H_*w*_ is tilted away from the WS_2_ surface while the other remains parallel (Figure 4d). Importantly, the orientation of O–H bonds that are perpendicular to the surface normal can facilitate lateral hydrogen bonding between water molecules. This clustering behavior, which promotes hydrogen bonding among water molecules, is clearly illustrated for one water layer in SI Figure S 1d. The reduced probability density at (90°, 90°) relative to other peaks suggests that interactions occur between the WS_2_ and H_2_O, rather than the water maintaining a flat, cyclic structure.

The two distinct peaks in three water layers for both H_*w*_ and O_*w*_ are at 2.8–3.0 and 5.8–6.1 Å. This indicates that at the surface of WS_2_ there is a distinct double-layer structure to the water. This is seen to an extent in the two-layer system as well, with peaks at 3.1–3.2 and 4.6–5.2 Å. There is a smaller peak centered around 10 Å for both H_*w*_ and O_*w*_ in the three-layer system. This may indicate a greater long-range structure at the WS_2_/H_2_O interface than originally anticipated. These peaks closely correspond to values calculated from AIMD of water on WS_2_, MoS_2_, and WeSe_2_, which show peaks at approximately 3, 6, and 10 Å for all three TMDs.^50,51^ Similar water structuring at the interface has also been observed for h-BN, and graphene.^52^

#### 3.1.3 SiO_2_/H_2_O/WS_2_ Interface

Finally, we investigate the static properties of the SiO_2_/H_2_O/WS_2_ interface. For one layer of water, the H_*w*_ and O_*w*_ peaks are very sharp (Figure 3e,3f), indicating strong structuring at the interface, with increased structuring compared to the broader SiO_2_/H_2_O initial peak. The positions of the most probable orientations in the confined interface closely resemble those observed in the SiO_2_/H_2_O JPD, indicating that similar orientations are favorable (Figure 4e). Notably, the dominant orientation in the confined system at (110°, 110°) is also seen in the unconfined SiO_2_/H_2_O, where the H_*w*_ is tilted downward toward the SiO_2_ surface. The two additional angles at (115°, 15°) and (15°, 115°) are equivalent and show the same orientation as in SiO_2_/H_2_O, with one H_*w*_ directed upward away from the SiO_2_ surface (Figure 4f).

However, in the two-layer system, there is no sharp initial O_*w*_ peak observed in the 1D profile, suggesting that either multiple orientations of water are favorable, or that the water is less structured. When we examine the JPD of water 4 Å away from the surface (the BIL), we observe that the localization of water angles changes for all interfaces. The SiO_2_/H_2_O interface remains the most consistent across different water layers, indicating that the BIL is tightly bound and less sensitive to changes in additional water (SI Figure S2). In contrast, for the WS_2_/H_2_O and SiO_2_/H_2_O/WS_2_ interfaces, the JPD differs from that of the one-layer systems. For both interfaces the two-layer water angle is (90°, 90°), thus, water is expected to predominantly orient parallel to the underlying substrate. A parallel orientation of water has been observed in a single layer of WS_2_/H_2_O; however, strong localization has not been detected within the confined one-layer system. The changes observed in the confined two-layer system may result from the increased availability of water, which facilitates the formation of larger hydrogen bonding networks among the water molecules. This effect is maximized by parallel orientations of water.

Investigating the WS_2_/H_2_O side of the confined interface, H_*w*_ is closer to WS_2_ than O_*w*_, as exhibited by the final peaks and shoulders from H_*w*_ in Figure 3e,f. This is the same behavior as seen at the unconfined WS_2_/H_2_O interface, highlighting that WS_2_ does have a small impact on the water structure. Further comparison of peak positions between SiO_2_/H_2_O and SiO_2_/H_2_O/WS_2_ reveals that they are aligned quite well, with only minor deviations (SI Table S1). We observe that the initial peak for one and two water layers is located closer to the SiO_2_ surface compared to the SiO_2_/H_2_O interface. In the case of three water layers, the alignment of the initial peaks in both interfaces becomes comparable, and they also exhibit similar width.

Three water layers shows further change in water structure at the confined system JPD (SI Figure S4c). This suggests that, while the layering away from the interface resembles that of the SiO_2_/H_2_O interface, the arrangement of confined water is distinct, showing increased variation in the orientation of water molecules as the number of water layers increases. This difference in water structure is also evident in the 2D profiles in Figure S1, where the SiO_2_/H_2_O water is more localized around the silanol groups compared to the confined water.

Overall, the orientation of confined water is primarily influenced by the SiO_2_ surface, with minimal contribution from WS_2_. For all interfaces, H_*w*_ is oriented toward the substrate, which has been shown to be energetically favorable at both the SiO_2_ and WS_2_ surfaces.^53^ Previous studies on SiO_2_ surfaces indicate that the number and type of silanol groups affect the behavior of water differently. Isolated silanol groups, which are used in our study, typically facilitate water approaching the SiO_2_ surface more closely. The confinement of water may exacerbate this, as seen in the 1D density profile and the differences in JPD. HD-VSFG and CMD calculations have demonstrated that water molecules with their hydrogen atoms pointing toward the surface are favored.^13,43,54^ Nevertheless, we acknowledge the possibility of additional orientations, as our sampling is limited due to the AIMD method, potentially resulting in less reliable intensities in the JPD graphs. Previous literature suggests a variety of orientations at the SiO_2_ interface.^55^

### 3.2 Dynamic Properties

We now turn to examining the dynamic properties of three interfaces, including hydrogen bonding and water mobility. These dynamic properties may provide insights into the reasons behind the observed structure and orientation of water.

#### 3.2.1 SiO_2_/H_2_O

**Figure 5:**
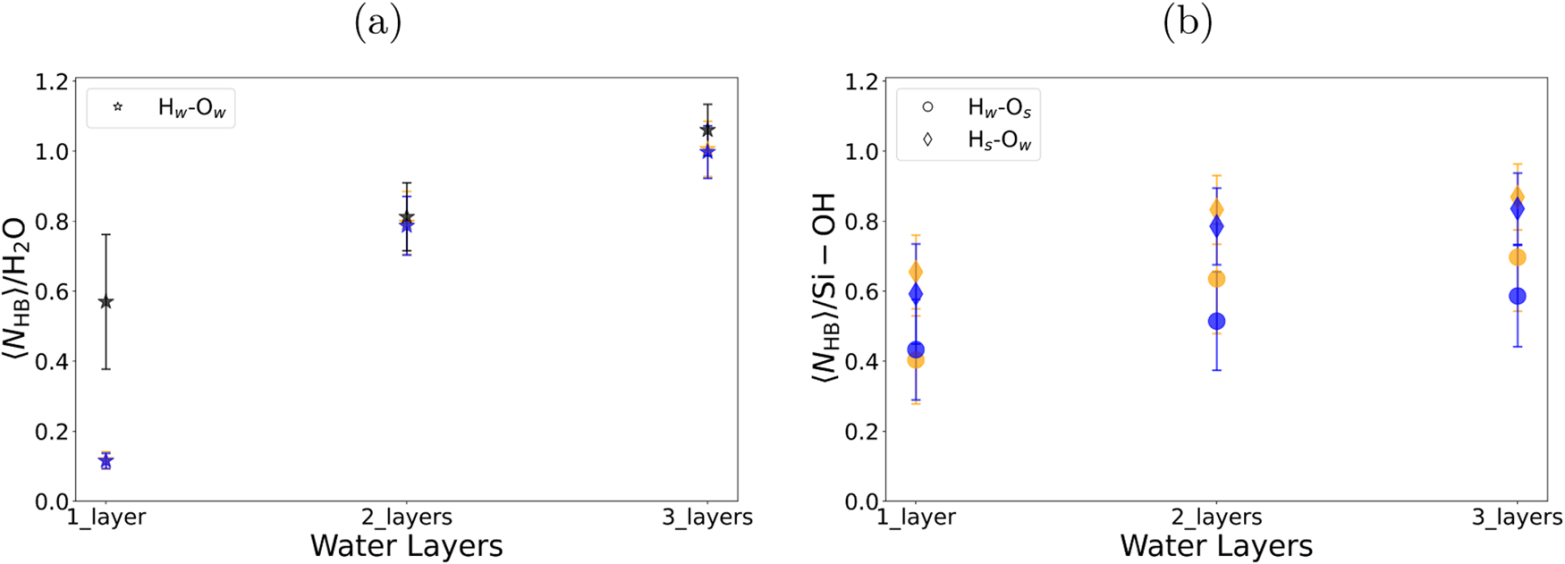
Average number of hydrogen bonds for different number of water layers, (a) Hbonding of water–water and (b) water to silanol groups HBonding. The HBonds to silanol H_*s*_ or O_*s*_ are w.r.t the total number of silanol groups, and the H_*w*_-O_*w*_ HBonds are w.r.t total number of water molecules. The different interfaces are shown as different colors: blue: SiO_2_/H_2_O, black: WS_2_/H_2_O, and orange: WS_2_/H_2_O/SiO_2_.

**Figure 6:**
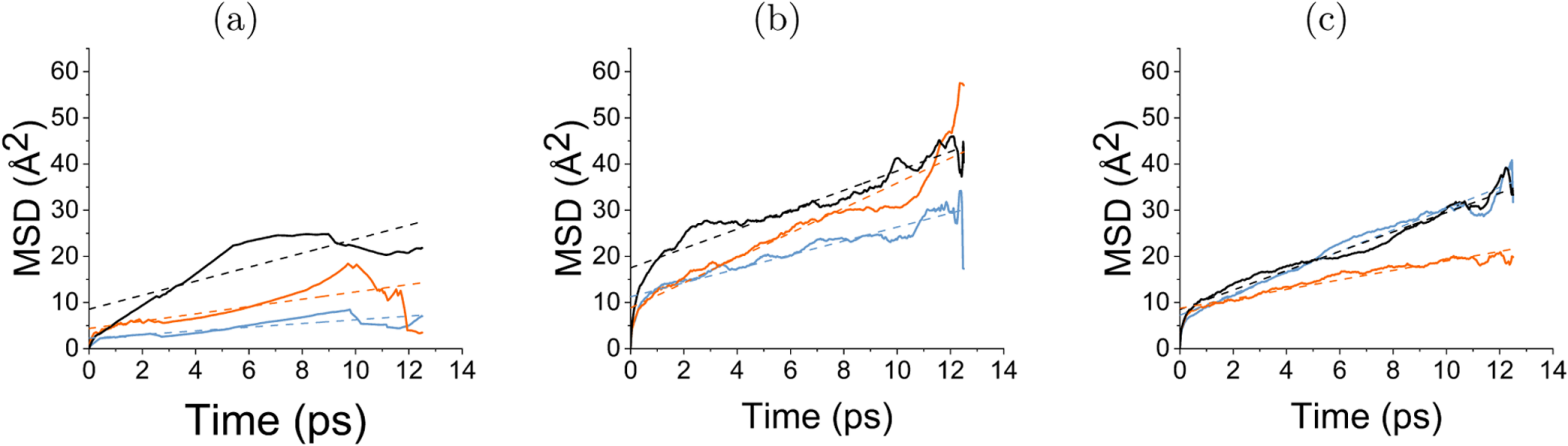
2D MSD in the XY direction for all interfaces based on the number of water layers. (a) 1-layer, (b) 2 layers, (c) 3-layers. The different interfaces are shown as different colors: blue: SiO_2_/H_2_O, black: WS_2_/H_2_O, and orange: WS_2_/H_2_O/SiO_2_. The linear fit of the data is shown by the dashed line corresponding to the material color.

For the SiO_2_/H_2_O with a monolayer of water, there is minimal water–water (H_*w*_-O_*w*_) HBonding, with an average of 0.12 HBonds/water molecule (Figure 5a). This value is significantly lower than the average number of HBonds formed between silanol oxygen -water hydrogen (O_*s*_-H_*w*_) or water oxygen - silanol hydrogen (O_*w*_-H_*s*_) which are 0.43 and 0.59 HBonds/silanol group, respectively (Figure 5b). The MSD analysis of SiO_2_/H_2_O (Figure 6a) shows that the single water layer exhibits the lowest displacement, with a diffusion coefficient (*D*) of 0.10 Å^2^/ps. This indicates that water molecules are strongly bound to the silanol groups, which limits their mobility. Therefore, at monolayer coverage, the silanol groups on the silica surface significantly disrupt the hydrogen bonding network (HBN) of water, a phenomenon also observed in previous CMD studies.^13^

As additional layers of water are introduced, the average number of H_*w*_-O_*w*_ HBonds increases, reaching 0.80 HBonds/water molecule for the two-layer system and 1.00 HBonds/water molecule for the three-layer system. Therefore, neither the two- nor the three-layer system achieves a fully saturated HBN.

**Figure 7:**
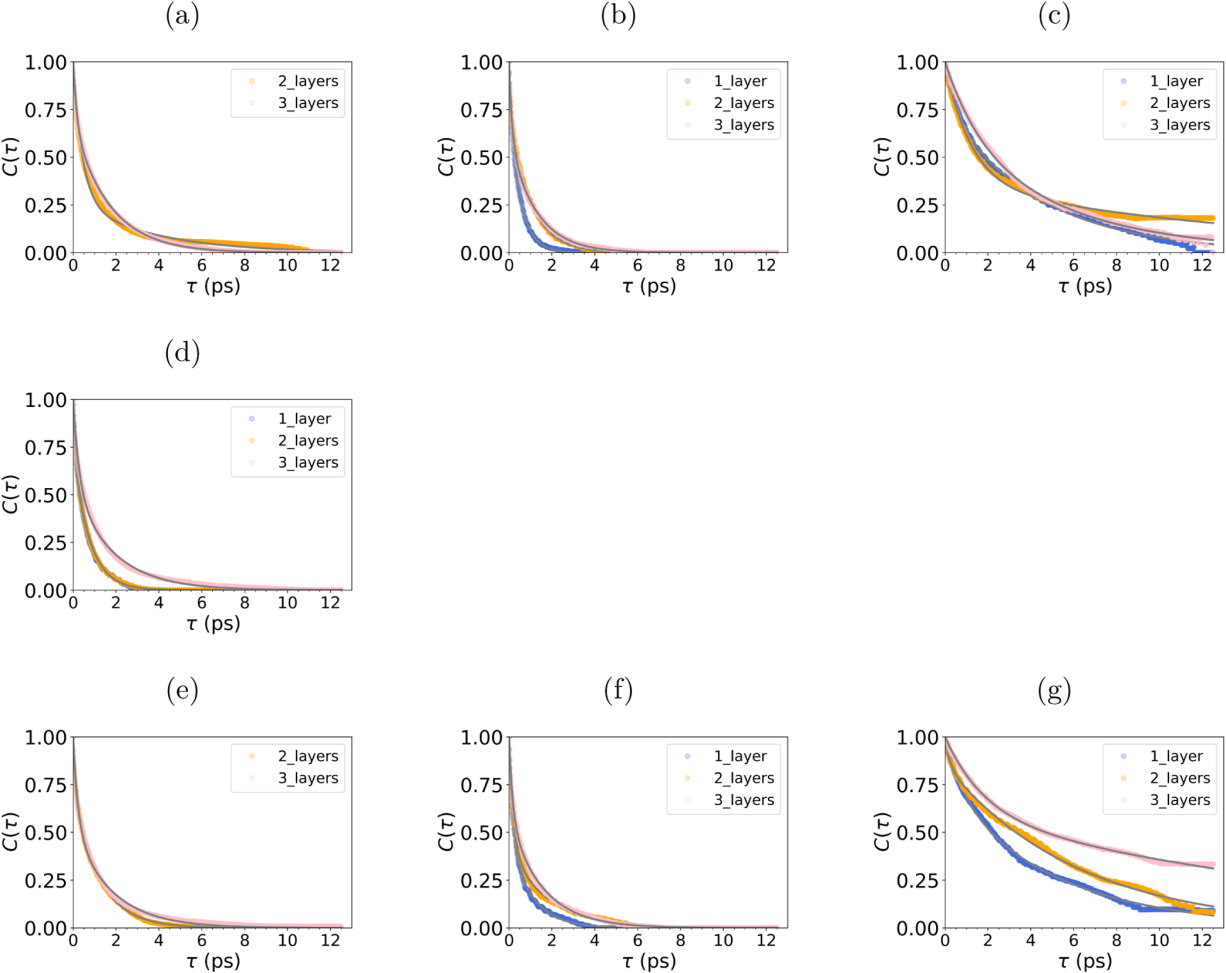
Hydrogen bonding autocorrelation for different layers of water in (a–c) SiO_2_/H_2_O, (d–f) SiO_2_/H_2_O/WS_2_, and (g) WS_2_/H_2_O. The first column (c, d, g ) is the autocorrelation of water–water hydrogen bonds (O_*w*_ – H_*w*_). The second column (b, e) is the autocorrelation for water hydrogen and silanol oxygen (O_*s*_ – H_*w*_). The final column (c, f) is the autocorrelation between water oxygen - silanol group hydrogen bonds (O_*w*_ – H_*s*_). The biexponential fit from which the HBond lifetime is derived is shown in gray.

The lifetimes of HBonds determined by the autocorrelation function show that O_*w*_-H_*w*_ HBonds last up to 1.31 (±0.020) ps in two layers, and 1.21 (±0.004) ps in three layers (Figure 7a). In one layer, the water does not form a sufficient number of HBonds to derive an accurate autocorrelation for the continuous HBond lifetime.

The O_*s*_-H_*w*_ HBond has a shorter lifetime of 0.43 (±0.001) ps in one layer, which increases to 0.75 (±0.002) ps for two layers and 0.81 (±0.003) ps for three layers (Figure 7b). In contrast, the O_*w*_-H_*s*_ lasts significantly longer than any other type of HBond (Figure 7c). The HBond lifetimes are 3.47 (±0.023), 6.12 (±0.098), and 4.08 (±0.056) ps for one, two, and three layers, respectively. As shown in the average number of HBonds in Figure 5b, there are more O_*w*_-H_*s*_ across all water layers. This suggests that these abundant and long-lived hydrogen bonds may contribute to the BIL water observed at the SiO_2_ interface.

The mobility of water *D* increases with the number of water layers, reaching 0.38 (±7.5 × 10^–4^) Å^2^/ps for two layers, and 0.58 (±6.9 × 10^–4^) Å^2^/ps for three layers. This increased mobility with additional water layers reflects the more liquid-like nature of water located farther from the silica surface, with weaker structuring by silanol groups. This increase in mobility with distance from SiO_2_ can be clearly seen in SI Figure S6a, where less-ordered, distant water layers contribute more significantly to the increased MSD. A similar reduction in mobility due to surface structuring by silanol groups has also been observed in water at the Al_2_O_3_ interface, as demonstrated by AIMD simulations.^56^

#### 3.2.2 WS_2_/H_2_O

Investigation of HBonding at the WS_2_/H_2_O interface reveals that the absence of direct HBonding between WS_2_ and H_2_O results in an average of 0.57 HBonds/water molecule (Figure 5a). The faster decay of HBond lifetimes for WS_2_/H_2_O, compared to SiO_2_/H_2_O, is evident in Figure 7d. In addition, the HBN at the WS_2_/H_2_O interface exhibits greater fluctuations than at other interfaces (SI Figure S5b). Therefore, HBonds break more quickly for systems with one and two layers of water, with a lifetime of 0.57 (±0.003) and 0.59 (±0.002) ps, respectively. The diffusion coefficient for one and two layers was calculated to be 0.38 (±1.8 × 10^–3^) Å^2^/ps and 0.53 (±1.1 × 10^–3^) Å^2^/ps, respectively. Overall, clustered water is mobile with HBonds breaking and reforming rapidly.

However, as shown in Figure 6a, there is a deviation from the linear fit of the MSD for one layer. Despite the self-diffusion error being low, the accuracy may be compromised due to the limited number of water molecules sampled. This deviation could be due to either the clustered nature of water or the reduced number of water molecules and short-time scale, which raises doubts about the exact diffusion coefficient values. This is underscored by the R-squared value for one layer of 0.65, indicating that the fit is not optimal. However, in SI Figure S6 that the movement of molecules within 4 Å from the WS_2_ surface is greater than at any other distance for all water layers, suggesting this may also be a common phenomenon at the interface rather than a sampling issue.

For the three-layer system, the HBond lifetimes increase to 1.14 (±0.010) ps, which is similar to the three-layer lifetime in SiO_2_/H_2_O. The HBonds are retained for longer periods creating a steady HBN, as seen in SI Figure S5. The MSD at both the WS_2_ and SiO_2_ interfaces converges (Figure 6b,c). This behavior suggests that the water dynamics at three layers resemble more bulk liquid/a DL than that of a BIL.

#### 3.2.3 SiO_2_/H_2_O/WS_2_

Similar to the SiO_2_/H_2_O system, the confined water SiO_2_/H_2_O/WS_2_ system shows minimal average H_*w*_-O_*w*_ HBonding of 0.12 HBonds/water mols for one water layer (Figure 5a). This bonding significantly increases to 0.80 in two layers and 1.01 in three layers. Therefore, the monolayer water HBN is disrupted by the silanol HBonding. The H_*w*_-O_*w*_ HBond lifetimes for two layers (0.89 ± 0.002) and three layers (1.06 ± 0.010) ps are shorter compared to those in the SiO_2_/H_2_O system, emphasizing this disruption (Figure 7e).

However, there are 0.13 more H_*s*_-O_*w*_ HBonds/silanol group in the confined one layer system compared to the unconfined system (Figure 5b). This further confirms the more rigid structure of water seen in the 1D profile of confined water compared to that of unconfined water, as the silanol group interactions are more dominant in the confined one-layer system.

For silanol-water HBonds in the confined system, the lifetimes of HBonds are the smallest for the one-layer system, with O_*s*_-H_*w*_ lifetimes lasting 0.57 (±0.005) ps (Figure 7f) and 3.99 (±0.029) ps for O_*w*_-H_*s*_ (Figure 7g). Although there is increased HBonding to silanol groups in the confined system, the *D* of 0.2 (±1.4 × 10^–3^) Å^2^/ps for the single water layer is greater than in SiO_2_/H_2_O. We suggest that this is due to HBond breaking and formation between different silanol groups, reflected in the increased dispersion of water density around these groups in the 2D density profile (SI Figure S1). Consequently, monolayer confined water may diffuse more readily between silanol groups.

The O_*s*_-H_*w*_ lifetime increases with the number of water layers, reaching 0.88 (±0.007) ps for two layers and 0.98 (±0.005) ps for three layers (Figure 7f). Similarly, the O_*w*_-H_*s*_ lifetimes show a similar trend, with significantly longer lifetimes of 5.30 (±0.019) ps for two layers and 12.04 (±0.103) ps for three layers, which approaches the simulation length. It is important to note that in both the SiO_2_/H_2_O and confined systems, the autocorrelation graphs for O_*w*_-H_*s*_ do not exhibit the rapid decay to zero observed in the other hydrogen bond lifetimes. This discrepancy may stem from the increased structuring of water influenced by H_*s*_, which increases the likelihood of water molecules exhibiting longer hydrogen bond lifetimes, thereby complicating the fitting process to a biexponential model. Furthermore, a significant issue is the reduced sampling resulting from the AIMD method, which increases the uncertainty of hydrogen bond lifetime values for O_*w*_-H_*s*_. As a result, these hydrogen bond lifetimes should be regarded with caution due to the accompanying increased error, as previously highlighted.

The MSD of the two-layer system shows an unexpected increase in *D* to 0.68 (±1.4 × 10^–3^) Å^2^/ps compared to the one layer. Regarding the HBond at the interface for two water layers, we note that the H_*w*_-O_*w*_ lifetime in the confined system is shorter than that in the unconfined system by 0.36 ps. Additionally, the two-layer O_*w*_-H_*s*_ lifetime is 0.97 ps shorter than in the unconfined system. Consequently, water is breaking HBonds more rapidly in the two-layer system which may contribute to the increase in MSD. This phenomenon may also reflect the change in the orientations of water in the confined two-layer system, which are parallel within 4 Å to the surface, as analyzed by JPD. The mobility is reduced to 0.27 (±4.6 × 10^–4^) Å^2^/ps in three layers. This reduction comes with an increased lifetimes of all types of HBonds in comparison to the SiO_2_/H_2_O system.

However, the nonlinear increase in total displacement between 10–12.5 ps in the two-layer system highlights issues with AIMD. The 12.5 ps time scale restricts the statistical reliability of dynamic data, while the small simulation box diminishes the movement of water molecules, potentially leading to the overrepresentation of anomalous data. These factors can result in longer HBond lifetimes and increased correlation between water molecules impacting the MSD.

Overall, for two and three water layers, the water remains relatively mobile in all systems. This reinforces the conclusion that these layers are not completely rigidly structured (even under confinement) and instead retain the ability to move and reorient.

## 4 Discussion and Conclusions

We used AIMD to provide the first insight into the behavior of different layers of water at the SiO_2_/H_2_O/WS_2_ interface. Currently, there are no experimental measurements available, as these surfaces are challenging to prepare and investigate, unlike mica. The confined water is structured by the SiO_2_ silanol groups, which cause the water to form a binding interfacial layer. When silanol groups are present alongside monolayer water, little consistent hydrogen bonding is observed between the water molecules; instead, water is predominantly hydrogen bonded to the silanol groups. This silanol-water HBonding interaction increases under confinement. In contrast, mica allows for the formation of an ice layer when water is confined with 2D materials, which relies on intact water–water hydrogen bonds. Confined water exhibits increased mobility for one and two water layers compared to SiO_2_/H_2_O, but decreased mobility for three water layers. Therefore, the water-silanol HBonding interaction is key to the properties of confined water at the SiO_2_/WS_2_ interface. Furthermore, the relative humidity during sample preparation influences the properties of the water, with increased RH represented by the three layers of water and low Rh by the one layer.

The introduction of WS_2_ to confine the water results in a slight restructuring of water, with water hydrogen atoms oriented toward the WS_2_. However, this effect is minimal when compared to the HBonding interactions with silanol groups. Additional layers of water result in reorientation of water close to the surface for both SiO_2_/H_2_O/WS_2_ and WS_2_/H_2_O interfaces.

To assess the accuracy of our AIMD results, we compare them to relevant values from the literature. We note that the diffusion coefficient calculated for SiO_2_/H_2_O is greater than in previous CMD work on the cristobalite surface of 0.028 Å^2^/ps.^13^ To further investigate this difference, we examine the MSD of water based on its position relative to the surface to see if *D* is reduced to a comparable value due to the HBonding between silanol and water (SI Figure S6a). The MSD is reduced within 4 Å from the surface, as expected. In the three-layer system, the MSD substantially increases at distances of 8–12 Å from the SiO_2_ surface. In order of increasing *D*, the water within 4 Å is one (0.10 Å^2^/ps) < three (0.28 Å^2^/ps) < two (0.35 Å^2^/ps) water layers. Therefore, we could not identify a reason for the magnitude of difference in D. AIMD may more accurately account for hydrogen bonds, allowing for improved modeling of movement. In contrast, CMD results, obtained over a longer time scale, provide greater statistics, indicating less movement near the SiO_2_ surface overall. Thus, additional work is required to accurately determine the MSD of water at the 10 1̅ cristobalite surface. We also note that an elevated temperature of 400 K was used in AIMD to mitigate overbinding of water, but this may contribute to increased water mobility.

The continuous lifetimes of HBonds reported in the literature for bulk H_*w*_-O_*w*_ range from 0.18 to 0.7 ps depending on hydroxylation and CMD force field.^57−60^ In contrast, the lifetimes of silanol-water hydrogen bonds are significantly longer, at 1.73 ps.^57^ In our work, the HBond lifetimes are markedly higher than those previously reported; specifically, the lifetime of O_*w*_-H_*s*_ is greater than anticipated. However, the shorter lifetimes of O_*s*_-H_*w*_ may have some impact, as prior studies have not distinguished between the different types of HBonding. Therefore, averaging of both types of silanol-water HBonds may lead to more comparable HBond lifetimes. Additionally, the water used in our simulations is not bulk, which may contribute to this difference, as the water molecules are not in the same environment in regards to the O_*w*_-H_*w*_ HBonds. However, we may also attribute this discrepancy to the short-time scale of the AIMD calculations, which cannot be performed on the same nanosecond time scale as CMD. Consequently, less dynamic data can be analyzed and averaged.

The bulk water *D* has been calculated to be between 0.172 and 0.591 Å^2^/ps, based on both CMD simulations and experimental literature.^13,61−65^ Our diffusion coefficients are at the higher end of this literature range for all interface systems, except for the confined water two-layer interface which exceeds the range. The WS_2_/H_2_O interface with three layers of water presents the most comparable result to bulk water. This system is less influenced by silanol groups and features only O_*w*_-H_*w*_ hydrogen bonds. The *D* of 0.58 Å^2^/ps remains within the literature range, suggesting that water mobility may be accurately represented in the AIMD simulation. However, as previously highlighted, future work to extend the time scale of the simulations may offer improved insight into the water dynamics of the system.

## Supporting Information Available

Table of 1D density peak values, 2D density profiles, JPD for two and three water layers for all interfaces, HBonds over time, MSD of water by distance to underlying substrate (PDF)
