# Supplementary material for: Structure and Dynamics of Water Confined at the SiO2/WS2 Interface
Source: J Phys Chem C Nanomater Interfaces. 2025 Feb 17;129(8):4261–71. doi: 10.1021/acs.jpcc.4c08392 (PMC11874030; doi:10.1021/acs.jpcc.4c08392)
Supplement: Supplementary file 1 — jp4c08392_si_001.pdf [file jp4c08392_si_001.pdf]

Supporting Information: Structure and Dynamics of Water Confined at the SiO_2/WS_2 Interface

Katherine L. Milton,*,† Laura Hargreaves,† and Alexander Shluger*,†,‡

†*Department of Physics and Astronomy and the London Centre for Nanotechnology, University College London, Gower Street, London WC1E 6BT, UK*

‡*WPI-Advanced Institute for Materials Research (WPI-AIMR), Tohoku University, 2–1-1 Katahira, Aoba-ku, Sendai 980-8577, Japan*

E-mail: katherine.milton.20@ucl.ac.uk; a.shluger@ucl.ac.uk

1D Density Profile Peaks

We briefly comment on additional SiO_2/H_2O features. Consistently, we observe in Table S1 that water in the WS_2/H_2O interface is located further from the surface compared to when SiO_2 is present. This highlights the influence of hydrogen bonds that draw water considerably closer to the SiO_2 surface.

In SiO_2/H_2O, a comparison of the O_w peak positions reveals that they are similar across all water layers, with only minor shifts in peak positions as the number of water layers increases (SI Table S1). However, the H_w peaks shift positions, and additional peaks appear when comparing three layers to two layers. Consequently, the layering of water, determined by the O_w atoms, likely remains consistent.

Table S1: Peak positions of the 1D density profile (Å) with shoulders given in brackets. The zero point from which the peaks originate corresponds to the top of the SiO_2 surface for SiO_2/H_2O and $SiO_2/H_2O/WS_2$ interfaces, and the closest sulfur plane for the WS_2/H_2O interface. Broad peaks are denoted with ^{b}.

Interface	No. Water Layers	Water Atom	P^1	P^2	P^3	P^4	P^5
SiO_2/H_2O	One	H_w	2.2 (3.2)				
		O_w	2.5				
	Two	H_w	2.4	4.0 (5.2)			
		O_w	2.7	4.7			
	Three	H_w	2.0	2.8	3.9	5.6	8.0
		O_w	(2.2) 2.7	5.5	7.3^b		
WS_2/H_2O	One	H_w	(2.2) 3.1				
		O_w	3.0				
	Two	H_w	(2.2) 3.1	4.0	5.2		
		O_w	3.2	4.6			
	Three	H_w	(2.0) 2.8	6.1	7.6	10.0^b	
		O_w	3.0	(5.2) 5.8 (7.0)	10.0^b		
$SiO_2/H_2O/WS_2$	One	H_w	1.9	3.0			
		O_w	2.2				
	Two	H_w	2.0	4.3 (5.5)			
		O_w	2.4^b	4.7			
	Three	H_w	2.0	3.6	5.5	(7.8) 8.8 (9.6)	
		O_w	(2.2) 2.8	4.9	7.2	8.9	

2D Density Profiles

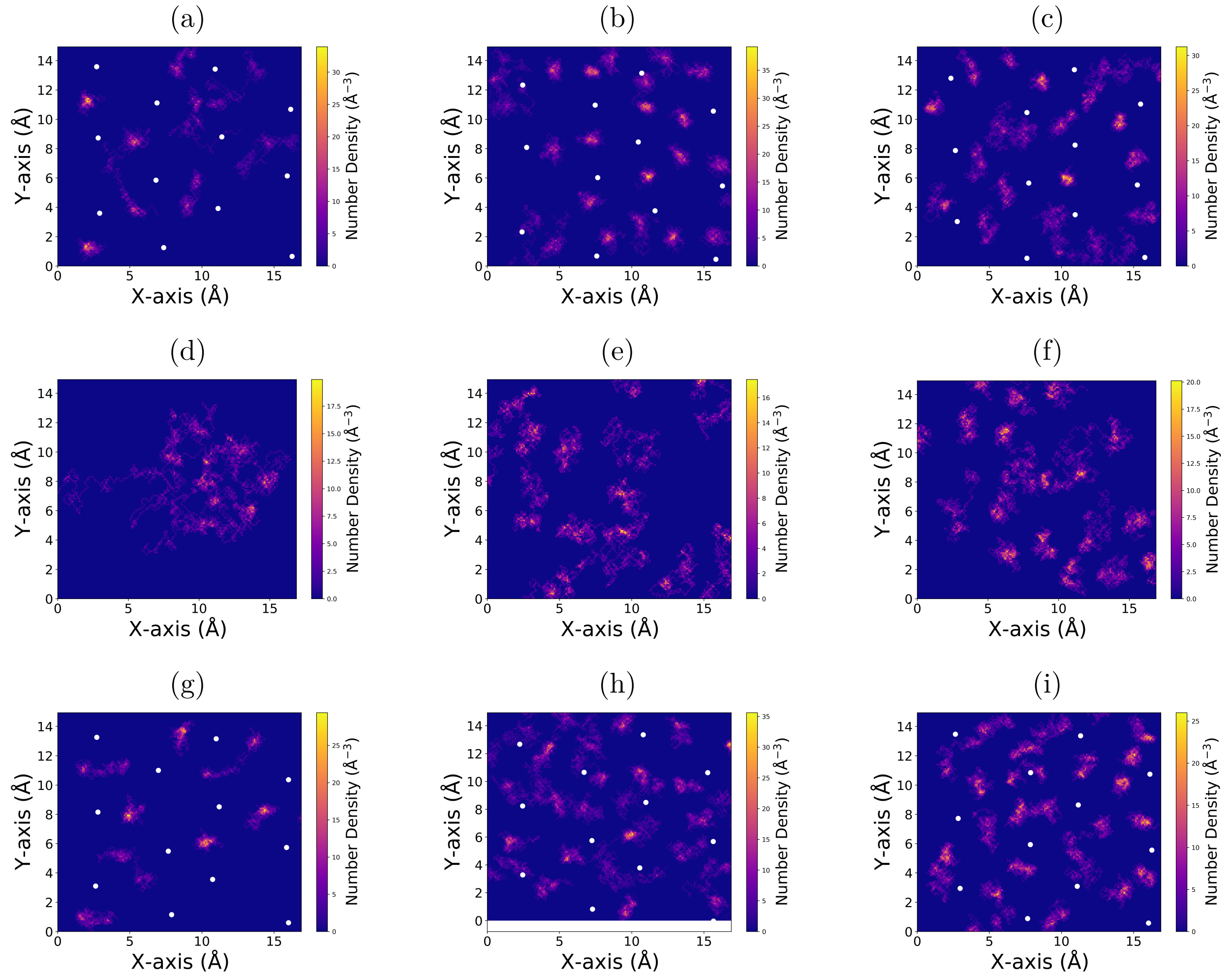

Figure S1: The XY 2D number density profiles of water on the underlying substrate. The interfaces are a-c) SiO_2/H_2O d-f) WS_2/H_2O, and g-i) $SiO_2/H_2O/WS_2$. The first column is for 1 water layer, the second column is for 2 water layers within 4 Å of the interface, and the third column is 3 water layers are also within 4Å of the substrate. For interfaces with SiO_2 present, the average position of silanol group oxygens on the surface are represented with white circles.

The 2D density profile is obtained using a similar method to the 1D profile, where the Cartesian axis is binned into length $\delta_x, \delta_y, \delta_z$. The 2D profile is calculated for each frame using

$$\rho_{xy}(x, y) = \frac{N_{xyz}}{\delta_z \times \delta_x \times \delta_y} \tag{S1}$$

and then averaging over the trajectory length. Here N_{xyz} is the number of water molecules within each box and the bins for all δ are 0.1 Å.

JPD 4Å From the Surface

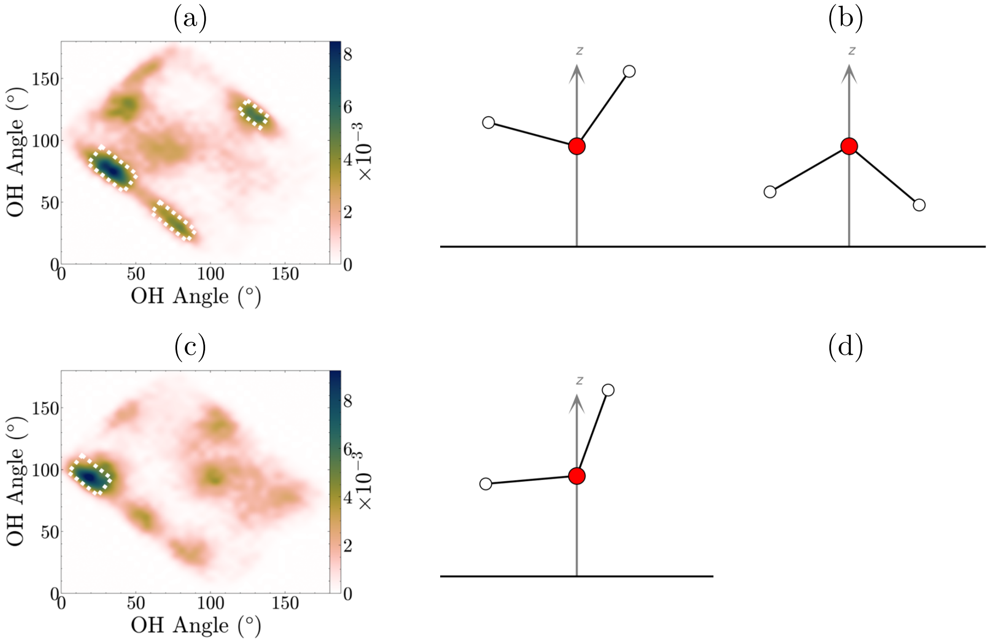

Figure S2: The probability distribution for water molecules 4 Å away from the SiO_2 surface at the SiO_2/H_2O interface. Water angles for **a-b)** two layers, and **c-d)** three layers of water. The first column (**a,c**) is the OH-OH angle for each system, the right-hand side (**b,d**) is the predicted orientations of water from the respective JPD graphs. The color bar shows the probability.

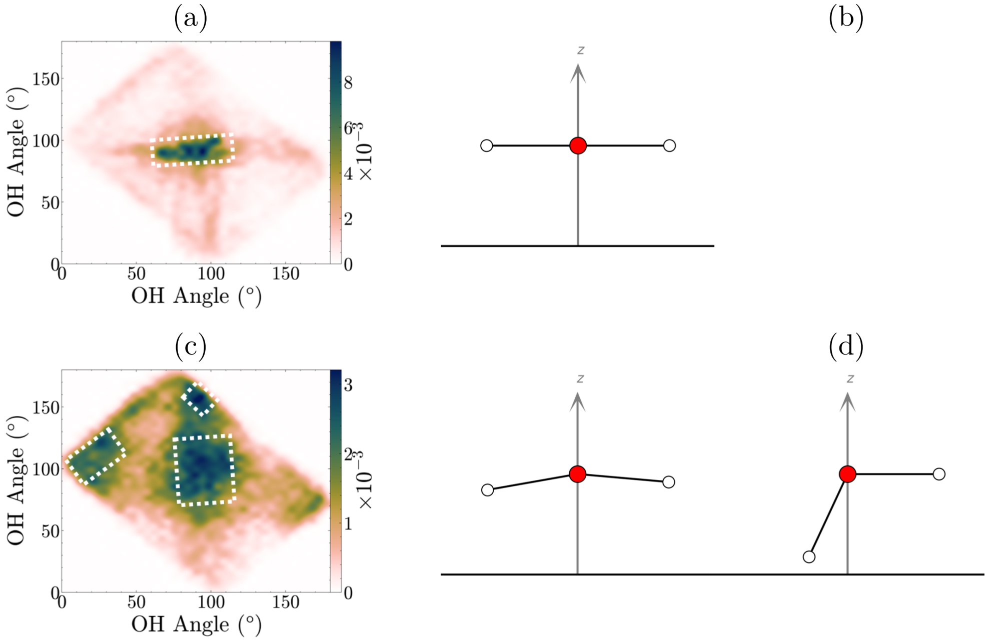

Figure S3: The probability distribution for water molecules 4 Å away from the WS_2 surface at the WS_2/H_2O interface. Water angles for **a-b)** two layers, and **c-d)** three layers of water. The first column (**a,c**) is the OH-OH angle for each system, the right-hand side (**b,d**) is the predicted orientations of water from the respective JPD graphs. The color bar shows the probability.

HBonds Over Time

The evolution of H_w-O_w HBonds over time indicates that at interfaces with SiO_2, HBonding is minimal for a single water layer. In the confined system, no HBonding is detected after 9 ps when using the geometric criteria. This highlights the reduced significance of H_w-O_w HBonds in water properties at one-layer coverage.

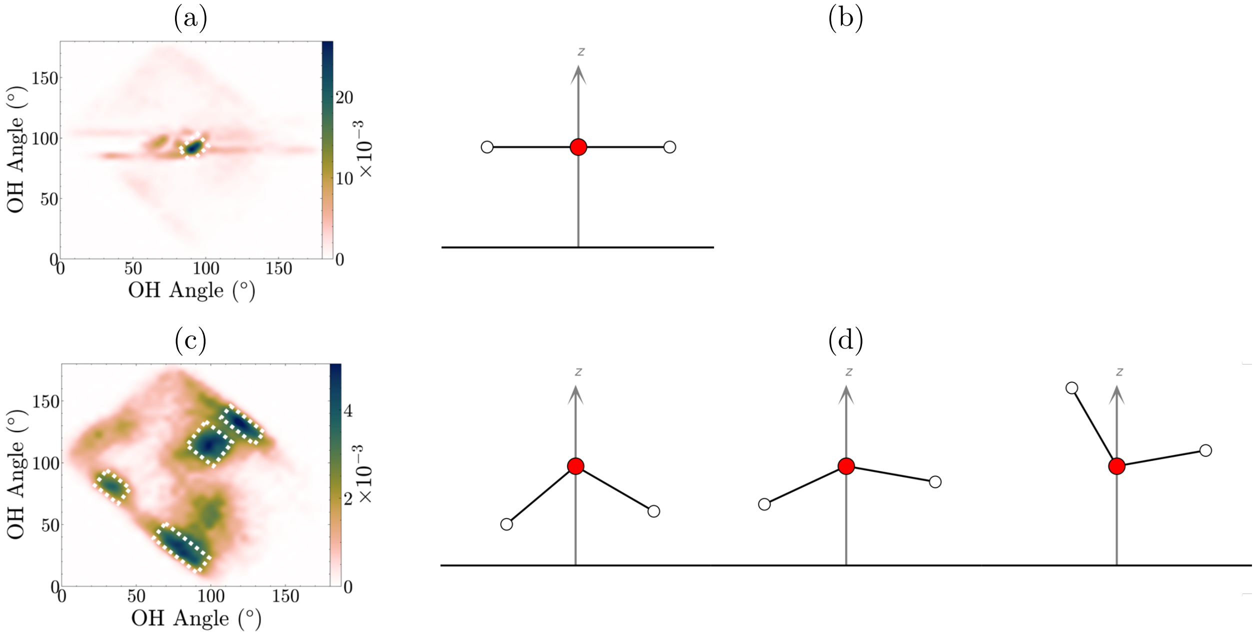

Figure S4: The probability distribution for water molecules 4 Å away from the SiO_2 surface at the $SiO_2/H_2O/WS_2$ interface. Water angles for **a-b)** two layer, and **c-d)** three layers of water. The first column (**a,c**) is the OH-OH angle for each system, the right-hand side (**b,d**) is the predicted orientations of water from the respective JPD graphs. The color bar shows the probability.

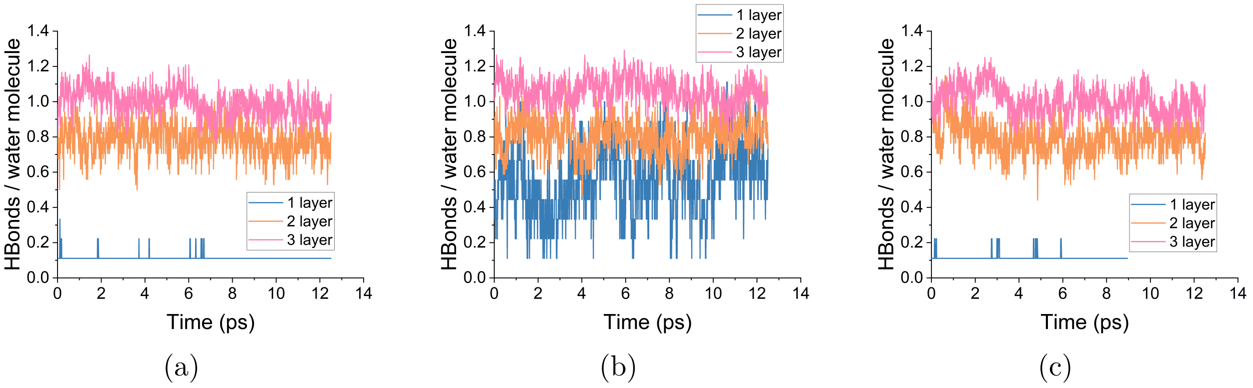

Figure S5: Hydrogen bonding over time of **a)** SiO_2/H_2O, **b)**WS_2/H_2O, and **c)**$WS_2/H_2O/SiO_2$.

MSD by Distance to Surface

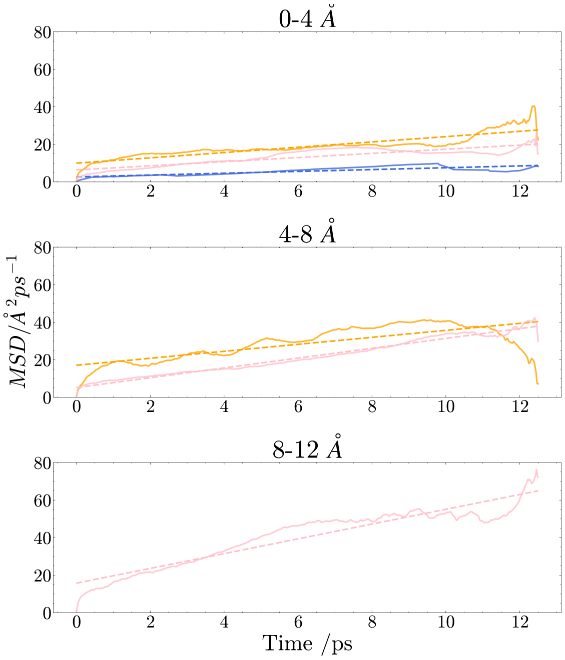

(a)

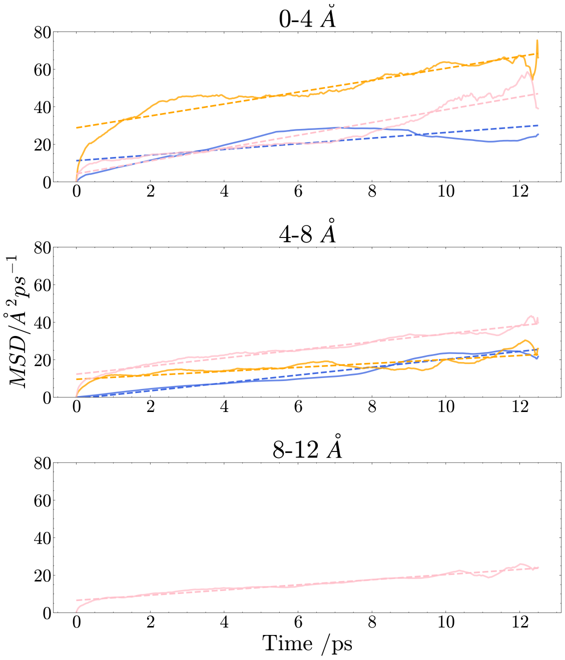

(b)

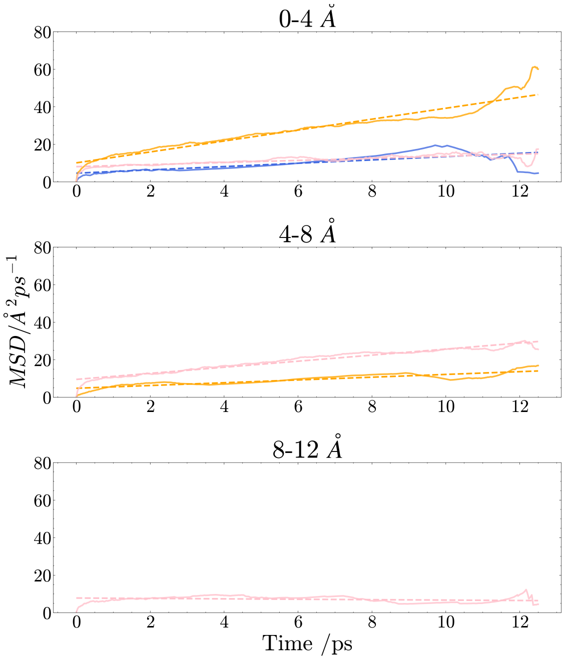

(c)

Figure S6: XY MSD for all interfaces split into the water distance from surface, the interface layers here are described by the water molecule density. **a)** SiO_2/H_2O,**b)** WS_2/H_2O and, **c)** $SiO_2/H_2O/WS_2$. The different water layers are given by the colors: one-layer: blue, two-layers: orange, three-layers: pink.
